# Giant root-rat engineering and livestock grazing activities regulate plant functional trait diversity of an Afroalpine vegetation community in the Bale Mountains, Ethiopia

**DOI:** 10.1007/s00442-024-05563-6

**Published:** 2024-06-01

**Authors:** Addisu Asefa, Victoria M. Reuber, Georg Miehe, Luise Wraase, Tilaye Wube, Nina Farwig, Dana G. Schabo

**Affiliations:** 1 Conservation Ecology, Department of Biology, Philipps-Universität Marburg, Karl-Von-Frisch-Straße 8, 35043 Marburg, Germany; 2 Vegetation Geography, Department of Geography, Philipps-Universität Marburg, Deutschhausstraße 10, 35032 Marburg, Germany; 3 Environmental Informatics, Department of Geography, Philipps-Universität Marburg, Deutschhausstraße 12, 35032 Marburg, Germany; 4 Department of Zoology, College of Natural and Computational Sciences, Addis Ababa University, Po Box 1176, Addis Ababa, Ethiopia

**Keywords:** Functional trait dispersion, Habitat filter, Habitat heterogeneity, Disturbance, Subterranean rodent

## Abstract

Disturbances from rodent engineering and human activities profoundly impact ecosystem structure and functioning. Whilst we know that disturbances modulate plant communities, comprehending the mechanisms through which rodent and human disturbances influence the functional trait diversity and trait composition of plant communities is important to allow projecting future changes and to enable informed decisions in response to changing intensity of the disturbances. Here, we evaluated the changes in functional trait diversity and composition of Afroalpine plant communities in the Bale Mountains of Ethiopia along gradients of engineering disturbances of a subterranean endemic rodent, the giant root-rat (*Tachyoryctes macrocephalus* Rüppell 1842) and human activities (settlement establishment and livestock grazing). We conducted RLQ (co-inertia analysis) and fourth-corner analyses to test for trait-disturbance (rodent engineering/human activities) covariation. Overall, our results show an increase in plant functional trait diversity with increasing root-rat engineering and increasing human activities. We found disturbance specific association with traits. Specifically, we found strong positive association of larger seed mass with increasing root-rat fresh burrow density, rhizomatous vegetative propagation negatively associated with increasing root-rat old burrow, and stolonifereous vegetative propagation positively associated with presence of root-rat mima mound. Moreover, both leaf size and leaf nitrogen content were positively associated with livestock dung abundance but negatively with distance from settlement. Overall, our results suggest that disturbances by rodents filter plant traits related to survival and reproduction strategies, whereas human activities such as livestock grazing act as filters for traits related to leaf economics spectrum along acquisitive resource-use strategy.

**Supplementary Information:**

The online version contains supplementary material available at 10.1007/s00442-024-05563-6.

## Introduction

Plants are a crucial component of ecosystems as they are the primary biomass producers and thus provide the energy that drives most ecosystem processes (Díaz et al. 2016). Thus, changes in plant communities often result in changes in important ecological processes, which in turn influence the extent, distribution, and diversity of organisms within ecosystems (Bernhardt-Römerman et al. 2011; Tessema et al. 2011; Díaz et al. 2016). Understanding the relationship of plant communities to environmental change caused by disturbances, e.g. bioturbation, and human activities, will allow projecting changes across trophic levels (Smith et al. 2022; Eldridge et al. 2023).

One important factor impacting plant communities is the activity of bioturbating animals. Especially subterranean rodents are known as ecosystem engineers due to their extensive underground tunnel digging and mound building that greatly alter soil properties (Reichman et al. 2002; Haussmann 2017) and impact plant communities by burying short vegetation and selectively feeding on preferred food plants (Jones et al. 1997; Escobedo et al. 2017; Asefa et al. 2022, 2023). On a landscape scale, they create a dynamic mosaic of burrow mounds varying in age, such as fresh active burrow mounds, old abandoned burrow mounds and mima-like mounds, characterized by different soil properties and plant communities (Jones et al. 1997; Cramer et al. 2014; Šklíba et al. 2017; Asefa et al. 2022, 2023). Consequently, engineering activities of subterranean rodents lead to increased microhabitat heterogeneity, which provides new resources such as open space and nutrients for species colonization and promote species coexistence within a community (Jones et al. 1997).

Similar to the engineering activities of rodents, human activities such as settlement establishment and livestock production also impact vegetation structure and plant community composition, mainly through grazing, trampling, defecation and urination (Dunne et al. 2011; Eldridge et al. 2016; Narantsetseg et al. 2018). For example, settlement establishment impacts plant community structure and composition directly by causing habitat loss and degradation via space use for house building. It also indirectly impacts plant communities due to increased activities, such as trampling (Bernhardt-Römerman et al. 2011), and due to its influence on the spatial movement and ranging patterns of livestock (Dunne et al. 2011; Asefa et al. 2023). The impacts of settlement establishment on plants, however, are more concentrated nearby settlements and declines as a function of increasing distance from settlement areas (Reitalu et al. 2010; Dunne et al. 2011; Asefa et al. 2023). Grazing and trampling by livestock reduces plant biomass, eliminates grazing intolerant species (Tessema et al. 2011) and damages seedlings and vegetative organs (Eldridge et al. 2016). At the same time, livestock activity creates open spaces for gap-colonizing plant species, promoting the dominance of unpalatable and grazing tolerant species (Tessema et al. 2011; Eldridge et al. 2016; Niu et al. 2019; Pavlů et al. 2019). Finally, livestock dung deposition and urination affect nutrient cycling and can reduce plant-species diversity by facilitating encroachment of exploitive native and/or non-native plant species (Pavlů et al. 2019).

The degree to which rodent engineering and livestock grazing impact plant communities is largely modulated by plant functional traits. Functional traits, one or a combination of traits related to a specific adaptation strategy, impact plant growth, reproduction and survival, and thus, modulate the tolerance to disturbances (Violle et al. 2007). As such, quantifying plant functional traits along disturbance gradients can reveal changes in functional strategies of a community (Grime 2001; Westoby et al. 2002), e.g. favouring species with fast growth and low competitive ability in highly disturbed sites (Smith et al. 2022). These changes in the functional composition of plant communities can occur even if taxonomic measures like species richness remain unchanged due to disturbance (Smith et al. 2022; Asefa et al. 2022, 2023). Thus, studying the functional composition is of importance to understand community drivers and mechanisms underpinning the changes.

A growing body of recent literature shows varying effects of different disturbance types on plant functional diversity and composition. For example, Choler (2005) has shown that the burrowing activity of the alpine marmot (*Marmota marmota*) benefits light-demanding rosette forbs, such as *Alchemilla* spp. In the alpine meadows of Qinghai–Tibet Plateau, Wang et al. (2020) have found negative associations between both plateau pika (*Ochotona curzoniae*) and plateau zokor (*Myospalax baileyi*) burrow densities and plant height. Similarly, an increase in burrowing activity of coruro (*Spalacopus cyanus*) leads to a decrease in plant functional diversity in an arid shrubland in Chile due to favouring a few invasive species (Escobedo et al. 2017). Compared with smaller seeds, larger seeds are known to survive longer in the soil, have better germination and improved seedling performance under stressful conditions (Suárez-Vidal et al. 2017). Thus, rodent disturbances may select for plants with larger seed size. Previous studies have also reported that over-grazing by livestock leads to a decrease in functional diversity by sorting for species with acquisitive traits, e.g. larger leaf area and higher nitrogen content, thereby leading to functional trait convergence (Deléglise et al. 2011; Narantsetseg et al. 2018; Wang et al. 2020). In the contrast, livestock grazing, via foraging and trampling, and human settlements, via intensive human trampling around villages, can reduce the abundance (biomass, cover) of dominant species (Bernhardt-Römerman et al. 2011; Dunne et al. 2011). These disturbances lead to changes in the competitive environment and availability of additional resources to support new species, thereby increasing functional diversity (Loucougaray et al. 2004; Koerner et al. 2018). Overall, findings of the above previous studies show that rodent engineering and human activities can act as habitat filters, and can potentially result in functional homogenization in species colonizing disturbed communities (Loucougaray et al. 2004; Choler 2005; Deléglise et al. 2011). However, increased habitat heterogeneity associated with these disturbances increases niche opportunities which may also lead to trait diversification within and between species of a community (Mayfield et al. 2010; Deléglise et al. 2011; de Bello et al. 2021).

The world’s oldest known human high-altitudinal residential site is located in the Bale Mountains (Ossendorf et al. 2019). This region is included in Conservation International’s Eastern Afromontane Biodiversity Hotspot. At the centre of these mountains is the Bale Mountains National Park (BMNP), which was inscribed on UNESCO World Heritage Site List on 18-Sep-2023 (https://whc.unesco.org/en/list/111/) and has been recognized as the single most important conservation area in Ethiopia (BMNP 2017). Despite the importance, similar to many other alpine ecosystems in Africa, more rapid ecosystem changes have been detected in the Bale Mountains over the past 40 years due to increasing settlement and agricultural activities, including livestock production (Tallents 2007; Johansson et al. 2014; BMNP 2017). Thus, the area is one of the best places to entangle the role of humans and other change drivers, such as rodent engineering, on biodiversity at high altitudes. Thus, understanding the interplay between disturbances from rodent engineering and human activities in impacting plant functional diversity is important to disentangle their relative role in shaping biodiversity patterns and to be able to predict what would happen in the future. Information derived from this understanding is important to enable informed decisions in response to changing intensity of the disturbances.

In this study, we evaluated the changes in functional trait diversity and composition of Afroalpine plant communities along gradients of engineering disturbances of a subterranean endemic rodent, the giant root-rat (*Tachyoryctes macrocephalus* Rüppell 1842), and human activities in the Bale Mountains of Ethiopia. For this study, we selected six traits to identify the main dimensions of variation in leaf and growth traits, which are known to have ecological functions related to resource use and acquisition, growth, survival and reproduction, and determined their association to root-rat engineering (fresh burrow density, old burrow density and presence of mima mound) and human disturbances (distance from settlement and livestock grazing intensity). Specifically, first we related multivariate functional trait diversity (functional divergence) to disturbance variables, and in a second step identified the association between each trait and disturbance. We tested two alternative hypotheses. First, we expected increased functional trait diversity with increasing root-rat engineering and human activities since both disturbances are expected to create habitat heterogeneity and increased resources (space and nutrients). In the contrary, if these disturbances act as habitat filters, we expected functional convergence along increasing disturbance gradients. If the second hypothesis would be correct, assuming that specific patterns of feeding and microhabitat changes are caused by rodents and livestock, we also expected disturbance specific associations with traits; specifically we expected strong association between giant root-rat and rosette (acaulescent, stoloniereous, rhizomatous) and larger seed mass traits, whilst human disturbance was expected to be associated with acquisitive traits, e.g. larger leaf area and higher nitrogen content.

## Materials and methods

### Study area

**Fig. 1:**
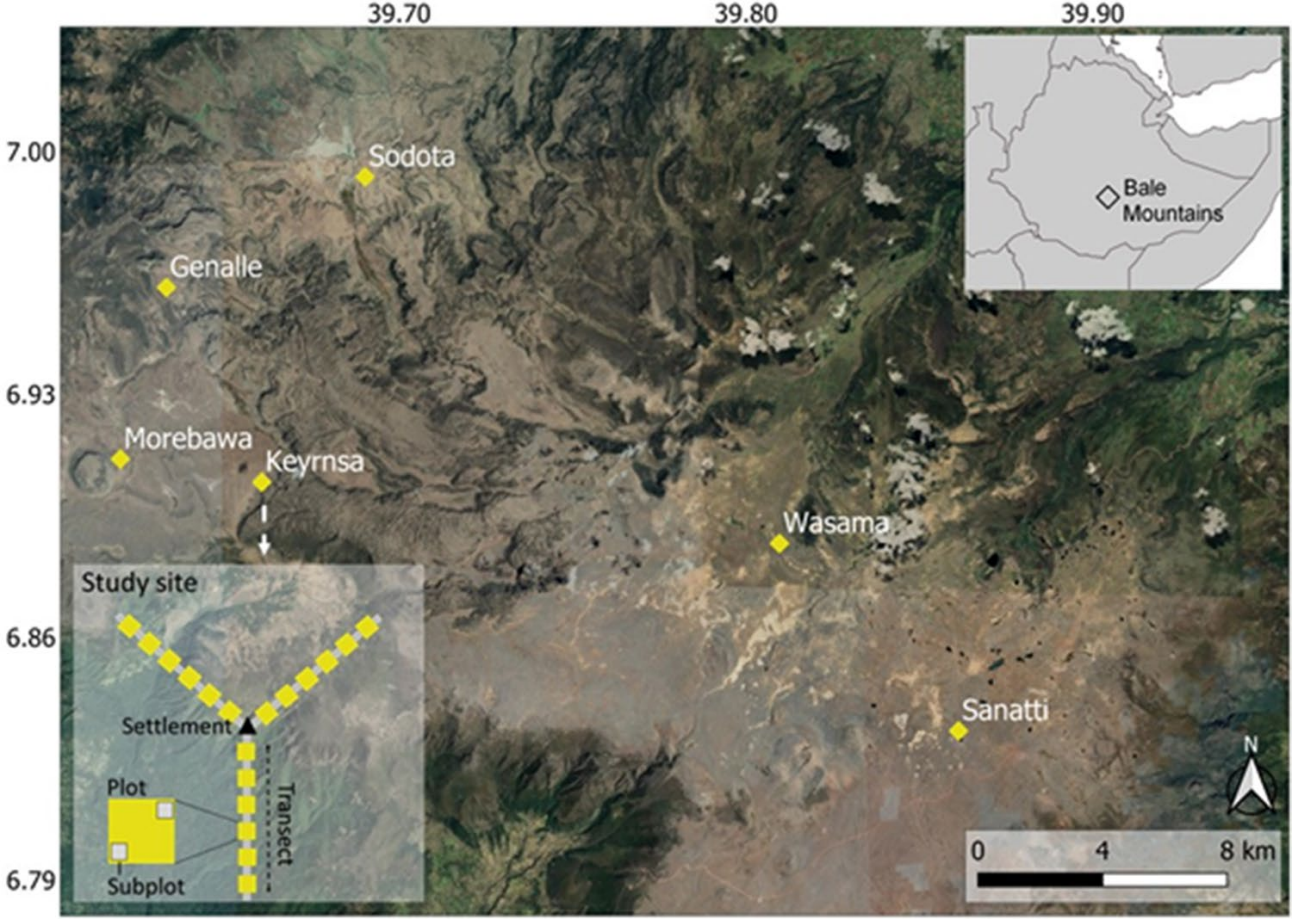
Overview map of the Bale Mountains National Park and its location in southeast Ethiopia (top-right inset) and the six study sites, and detailed inset map (bottom-left) showing the set-up of one study location with three transects of 1.5 km length, six study plots along each transect and two subplots within each plot (for detailed description see Methods section “Study area”)

This study was conducted in the Afroalpine ecosystem of the Bale Mountains National Park in South-Eastern Ethiopia (BMNP; 6.508–7.178N, 39.508–39.928E; Fig. 1; Asefa et al. 2022, 2023) between February and March 2021. The Bale Mountains represent the largest area of Afroalpine vegetation over 3000 masl in Africa (Yalden 1983). Elevation in the Bale Mountains ranges between 1500 and 4377 m asl. The area experiences two rainy seasons, with lighter rains from March to June and heavy rainy season from July to October, and a dry season between November and February; mean annual rainfall is approximately 1000 mm (Miehe et al. 1994). The lowest and maximum recorded temperature in the Bale Mountains is −15 °C and 26 ºC, respectively (Miehe et al. 1994; BMNP 2017). The soils in the Bale Mountains, which are volcanic in origin and mainly derived from the basaltic and trachytic parent rock, are fairly fertile silty loams of reddish-brown to black colour (Hillman 1986; Miehe et al. 1994). Vegetation types of the Bale Mountains’ Afroalpine ecosystem include open grassland, grassland dotted with *Artemisia afra* shrub*, Helichrysum spp.* dwarf-scrub*, Alchemilla spp.* meadow*, Lobelia rhychopetalum*, and wetlands, such as alpine lakes, rivers, swamps and seasonal wetland grasslands; Tallents 2007; Asefa et al. 2022, 2023).

Similar to many other alpine ecosystems in Africa, more rapid ecosystem changes have been detected in the Bale Mountains over the past 40 years (Tallents 2007; Johansson et al. 2014; BMNP 2017). Subsistence livestock farming is one main income for the people and number of livestock, including cows (*Bos taurus*), horses (*Equus ferus*), sheep *Ovis aries*) and goats (*Capra aegagrus*), is estimated at 200–300 heads km^−2^ (Vial et al. 2010). Reber et al. (2018) have recorded a total of 870 settlements [houses] in the Afroalpine zone of the Bale Mountains. About 863 households, each having an average of four people, dwell in the study area (Asefa et al. 2023). For this study, we considered distance from settlement (a small village containing 10–150 houses) and livestock dung abundance as proxies for overall human activities. Some settlements are permanent and used throughout the year, whilst others are seasonal and used only during wet season, from April to August, when livestock are moved from lower pastures where agricultural crops are being grown (Asefa et al. 2023). Although most permanent and seasonal settlements were established before 30 years ago, the number of new settlements has been on the rise in the last three decades (BMNP 2017). The region is included to the exceptionally high diversity, rarity and endemicity of floral and faunal species but the ever-increasing human and associated settlement and livestock encroachments.

### Giant root-rat and its disturbances

The giant root-rat (*Tachyoryctes macrocephalus* Rüppell 1842), a subterranean rodent, is one of the several small mammal species restricted to the Afroalpine belt of the Bale Mountains (Yalden 1975). The species is restricted to < 1000 km^2^ area at altitudes from 3000 to 4150 m asl (Šumbera et al. 2018), where it is the main prey of the endangered Ethiopian wolf and numerous raptor species, such as golden eagle (*Aquila chrysaetos*), lesser-spotted eagle (*A. pomarina*), tawny eagle (*A. rapax*), Verreaux’s eagle (*A. verreauxi*) and augur buzzard (*Buteo rufofuscus*) (Sillero-Zubiri et al. 1995; Asefa 2007). The giant root-rat is a diurnal species and occurs with a density of 22–90 animals ha^−1^ (Yalden 1985; Sillero-Zubiri et al. 1995; Tallents 2007).

Giant root-rats construct extensive large underground burrow systems. An individual root-rat burrow system extends up to 34 m, and branches into short tunnels that comprise nesting and food caching and defecation chambers (Beyene 1986; Sillero-Zubiri et al. 1995; Yaba et al. 2011). Giant root-rats produce three types of burrow marks: fresh burrows, old burrows and mima mounds. Fresh burrows are easily distinguished from old burrows in that the former are freshly open or plugged holes that are currently active. In contrast, root-rat old burrows are abandoned burrows, with holes open or plugged with weathered soil, partially or wholly covered by vegetation regrowth, and sometimes occupied by other small rodents. Mima mounds are rounded dome-shaped structures formed by continually burrowing activities of giant root-rats that measure up to 27 m in diameter and 1.5 m in height (Beyene 1986; Sillero-Zubiri et al. 1995; Cramer et al. 2014; Šklíba et al. 2017; Wraase et al. 2023). Areas around root-rat mima mounds, which are giant root-rats’ favourite habitats, are characterized by the predominance of bare soil, as they eject soil from their burrow systems when excavating, and when plugging their burrow holes at night for thermoregulation (Yalden 1975). They also graze and gather vegetation for bedding around burrows, which further denudes the landscape (Beyene 1986; Yaba et al. 2011). As a result, giant root-rats have been known to cause changes in plant-species diversity and composition (Tallents 2007; Šklíba et al. 2017; Asefa et al. 2022, 2023). Thus, we determined the effect of giant root-rats on vegetation by measuring their burrow density.

### Experimental design

To examine the relationships between human settlement, livestock grazing, giant root-rat burrow density and plant functional traits, we relied on data on human activities, root-rat burrow density and plant-species abundance used in our recent publication (Fig. 1, Asefa et al. 2023). We systematically selected six study sites, between 5 to 20 km apart, spanning across the entire distribution range of the giant root-rat in alpine area above 3400 m asl. At each site, we selected two adjacent settlements (3–5 km apart)––one permanent settlement, and one seasonal settlement which occur in the wetter months, from April to August (Hillman 1986; BMNP 2017). All the selected settlements were known to be established 30 years ago (Hillman 1986; BMNP 2017), to avoid the influence of settlement age on the results due to their cumulative effects. Starting at the centre of each settlement, we established three 1.5 km long transects at an angle of 80–120° (see the inset map on Fig. 1). Along each transect, we established six 25 × 25 m^2^ plots at a distance of 250 m from each other. In total, there were 216 plots covering an area of 13.5 ha (see Asefa et al. 2023).

### Data collection

We undertook data collection in February and March of 2021 [for details on Methods of data collection see Asefa et al. (2023)]. At each plot, we recorded (1) rodent engineering activities measured as fresh burrow density (number plot^−1^), old burrow density (number plot^−1^) and presence-absence of mima mounds; (2) human activities measured as distance from settlement and abundance (number plot^−1^) of livestock (i.e. cows and horses) dung; and (3) plant species and species-specific cover.

We recorded plant-species identity and species-specific cover data within two 10 × 10 m^2^ subplots established at opposite corners of each plot. In each of these subplots, we identified all plants to species level, except grasses which were collectively recorded as a single morpho-species, and visually estimated, to the nearest 5%, the cover of each species. For analyses on the plot level, we averaged cover values of each species obtained from the two subplots. Seven species which occurred only in one plot, and about five grass species which were recorded as a single morpho-species were excluded from analysis. Thus, our data finally contained 61 species recorded across 216 plots (Asefa et al. 2023). Excluding grasses from analysis may potentially bias the functional trait diversity and trait composition values. However, we could not avoid this potential bias because many grasses were overgrazed and difficult to identify at species level during our survey and to compile species-specific trait values.

### Plant trait selection and data

Detecting significant associations between disturbance and functional traits depends on the list of examined traits (Westoby et al. 2002). Generally, a few traits, such as seed mass, leaf size and plant height, have been known to serve as surrogates for setting plant strategies related to the fundamental processes of plant life, i.e. dispersal, establishment, and persistence (Grime 2001; Westoby et al. 2002). As such, based on data availability we selected six plant traits (for which data were available) which are known to have ecological functions related to resource use and acquisition, growth, survival and reproduction and thus influence species responses to environmental changes caused by biotic disturbances (Díaz et al. 2016). These traits were: 1) adult plant maximum height (i.e. vegetative part; cm), 2) leaf area (mm^2^), 3) stem shoot-growth form, three categories: acaulescent (without aboveground stem), prostrate or erect, 4) dispersal mode, in three categories: seed alone, seed and rhizome and seed and stolones, 5) leaf nitrogen content (mg g^−1^), and 6) seed mass (mg). Species-specific trait data are provided in Online Resource 1.

Adult plant height is a measure of whole plant size and indicates ability to pre-empt resources, and therefore outcompete other plants. It also relates to plant resistance to damages from herbivory and burrowing activities of subterranean rodents (Díaz et al. 2016), with plants shorter than root-rat’s burrow mound height (upto 50 cm; our own unpublished data) being most susceptible to rodents’ disturbances. Stem shoot-growth form can also be linked with plant mechanical strength and resistance to biotic filters (Díaz et al. 2016). We extracted information on species-specific maximum plant height and stem shoot-growth form from botanical descriptions provided in the flora of Ethiopia and Eritrea (full references are provided in the Online Resource 2). Species taxonomic names mentioned in the text are following these flora books.

Leaf area (LA), one-sided surface area of an individual lamina, is a measure of leaf size and is relevant for light interception and has important consequences for leaf energy and water balance (Schrader et al. 2021). For leaf area estimation, we extracted information on minimum and maximum sizes of leaf length (L; average value) and leaf width (W; average value) of the leaf blade (i.e. excluding petioles), as well as information on leaf-shape type, from the flora of Ethiopia and Eritrea (the raw data is provided in Online Resource 3). In the case of compound leaves, single leaflets were treated as analogous to simple leaves with the exception of highly dissected pinnae for which we used the entire pinnae. Then, we estimated LA using Montemgory formula: Leaf area = cLW, where c is a correction factor to account for differences in leaf-shape type amongst species (Schrader et al. 2021). We used *c* values, ranging between 0.55 and 0.79, reported by Schrader et al. (2021), as their analysis is based on global level datasets that encompass larger number of species and all of the ten leaf-shape types we identified within our community samples (see Online Resource 3). In some cases, a species is characterized by having an intermediate shape between two shape types, e.g. elliptic to orbicular, in which case we used average values.

Leaf nitrogen mass is directly related to photosynthesis and respiration and reflects a trade-off between two different costs that increase with higher nitrogen; namely, the potential of suffering more herbivory (because of acquiring more nitrogen), on the one hand, and the greater photosynthetic potential that higher nitrogen allows, on the other hand (Díaz et al. 2016). For 54 species, we compiled data on leaf nitrogen content from the TRY Plant Trait Database30 (Kattge et al. 2022; https://www.try-db.org/TryWeb/Data.php, accessed 20-Feb-2023), and for 7 species for which traits are not included in the TRY database we extracted from published literature (see Online Resource 2).

Seed mass (mass of an individual seed plus any additional structures that assist dispersal and do not easily detach) indexes species along a dimension describing the trade-off between seedling competitiveness and survival on the one hand, and dispersal and colonization ability on the other (Thompson et al. 1993). We compiled data on seed mass for all species from Seed Information Database (SER, INSR et al. 2023; https://ser-sid.org). Similarly, vegetative dispersal is an adaptation to resist to/recover from herbivory damages and an adaptation strategy to compensate the erratic seed production and seedling establishment in alpine habitats (Choler 2005). We compiled information on vegetative dispersal from flora of Ethiopia and Eritrea (list of references are provided in the Online Resource 2).

For each species described in the flora of Ethiopia and Eritrea, information on its synonyms, subspecies or local variety is provided. Similarly, species names and their synonyms are also used in the TRY database. Thus, species names were standardized according to The World Flora Online database (www.worldfloraonline.org; accessed April 2023), and each species and its synonyms, subspecies or local variety is represented by a single value for each trait. The number of observations per trait and species range from a single one to hundreds. Thus, we calculated the geometric mean of all the records of a trait compiled. All data were unit-standardized and subjected to error detection and quality control. Accordingly, we excluded trait records measured on juvenile plants and on plants grown under non-natural environmental conditions, duplicate trait records for the same species on a particular trait, and potential outliers––trait records with a distance of > 3 standard deviations from the mean of the species (Díaz et al. 2016). The remaining dataset was used to calculate species mean trait values.

There are, at least, two closely related main caveats with our trait data, which may potentially influence interpretation of the results. The first main caveat is the missing of some important leaf traits, in particular the leaf mass area or the dry matter content of the leaves, since data values for these traits were not available for most of the species studied. The small number of leaf traits used in our study could thus have potential consequences for interpretation of the results. The second main caveat relates to using database traits. Intraspecific trait variation is extremely important in response to herbivory, and more generally to disturbance (Díaz et al. 2016). Thus, deriving trait values from a database to explore functional responses to disturbance is usually problematic for quantitative traits that show large intraspecific variation, and this may lead to missing a significant proportion of how the community responds (Kattge et al. 2022). It is possible that leaf traits in Bale might considerably differ from averages due to herbivory and other environmental factors, but we could not directly measure traits in the field due to logistic constraints. Thus, in the absence of detailed field data, we used averaged data across large scales as a first approximation.

## Statistical analysis

To assess whether increased giant root-rat disturbance and human activities act as agents creating habitat heterogeneity and thus increasing functional divergence or as a habitat filters by decreasing community functional divergence, we calculated functional dispersion (FDis) of all traits together based on principal coordinates analysis (PCoA) of a Hill-Smith dissimilarity matrix (FDis; Dray and Dufour 2007). FDis is the mean distance of each species to the centroid of all species in the community, weighted by its cover-abundance. Thus, a decrease in FDis means that community composition has shifted towards species that are more similar to each other, i.e. functional convergence, in response to increased disturbance. We used FDis because it is independent of species richness and takes into account species abundance; moreover, it can be used for multiple traits, as well as for continuous and categorical trait values (Dray et al. 2007). We calculated FDis using ade4 R package (Dray et al. 2007).

To evaluate whether the observed FDis of traits in each plot was higher or lower than expected from a null model, we estimated the standardized effect size (SES_FDis_) using 9999 randomly generated null communities with an ‘independent swap’ algorithm (Gotelli 2000). SES_FDis_ was calculated using the R package picante (Kembel et al. 2010) as the differences between the observed FDis and null FDis divided by the standard deviation (s.d.) of the null data; positive and negative SES_FDis_ values indicate higher or lower functional divergence than the null expectation values, respectively (Gotelli 2000; Kembel et al. 2010). In terms of assembly processes, positive SES_FDis_ values are considered to be caused by competitive exclusion that limits functional similarity or by habitat heterogeneity that increases resources and thus reduces competition, negative SES_FDis_ values can be caused by habitat filtering of functionally similar species or by strong competitive exclusion that leads to dominance of species with a specific trait, and values near zero indicates random distribution of traits or concurrent occurrence of competition or habitat heterogeneity and habitat filtering, off-setting their effects (Edwards et al. 2021). Thus, we tested whether mean SES_FDis_ in the 216 plots was significantly different from zero using *t* test. To determine the relationship between SES_FDis_ values with the giant root-rat and human disturbances, we also performed regression analysis based on GLMMs using glmmTMB package (Brooks et al. 2017), by including transect nested within site as random component. We checked for multicollinearity amongst predictors using the ‘performance’ R package (Lüdecke 2021); this confirmed weak collinearity between disturbance variables (VIF ranged between 1.11 and 1.73). We also used diagnostic plots in the DHARMa R package (Hartig 2021) and confirmed that model assumptions were met. As we did not find effects of settlement type (seasonal vs permanent) on plant functional composition and diversity, we assumed that both permanent and seasonal settlements similarly shape/modulate plant communities and did not report this in the rest of sections.

To directly measure the link between species traits and environmental data (the disturbance variables), we used Dray et al. (2008) novel version of the fourth-corner analysis provided in the ade4 package (Dray et al. 2007). Accordingly, we first conducted separate ordinations of three tables: ordination of table L (species abundance), which was done by a correspondence analysis (CA); table R (disturbance variables), done by a hillsmith function with row weights of table L; and table Q (species traits), done by a hillsmith function with the column weights of table L (Dray et al. 2007, 2008). To evaluate the global, or their joint multivariate relationship, significance of the trait-environment relationships, we conducted RLQ analysis using outputs of the ordinations described above. The RLQ analysis is a three-tables co-inertia analysis that tends to maximize the covariance between the sample site scores constrained by the environmental variables of table R and the species scores constrained by the traits of table Q (Dray et al. 2008). Finally, we undertook the fourth-corner analysis. In the fourth-corner procedure, a matrix L with species abundances is related to a matrix R with variables describing the extent of giant root-rat and human disturbances at the sample plots and a matrix Q describing species traits (Dray et al. 2007). The environmental matrix (R) contained the three root-rat disturbance variables: presence/absence of mima mound, root-rat old burrow density and root-rat fresh burrow density, and the two human activities: distance from settlement and livestock grazing intensity. The trait matrix (Q) was composed of six species traits: four continuous variables––plant adult height, leaf area, leaf nitrogen content and seed mass, and two categorical variables: stem-growth form (acaulescent, erect, and prostrate), and mode of propagation (seed only, seed and rhizome, and seed and stolones). We used the permutation model 6, with 999 permutations (Dray et al. 2008), which permutes all species within an entire column and row of the L matrix to test the null hypotheses of the observed pattern would be different from random. To evaluate the joint multivariate relationship, or the global significance of the traits-environment relationship we applied a multivariate test using fourthcorner2 function of the ade4 package. The significance of observed statistic was tested based on 999 Monte-Carlo permutations (Dray et al. 2007). To measure the strength and significance of the links between individual trait and environmental variable, we used a Pearson correlation coefficient for two quantitative variables, a Pearson Chi^2^ for two qualitative variables and a Pseudo-F for one quantitative variable and one qualitative variable (Dray et al. 2007). We conducted all analyses in the R environment (R Core Team 2020), and all R scripts used for analysis are provided in Online Resource 4.

## Results

Leaf area (LA) of plant species ranged between 5.6 and 4,0095 mm^2^ (mean ± SE: 2442.45 ± 835.30 mm^2^), whilst leaf nitrogen content (Nmass) ranged between 7.53 and 54.82 mg g^−1^ (mean ± SE: 24.26 ± 1.25 mg g^−1^). Adult plant height ranged between 5 and 300 cm (mean ± SE: 68.27 ± 8.84 cm), and seed mass between 0.02 and 1477 mg (mean ± SE: 1.15 ± 0.27 mg). Over half (38 species; 62.3% of the total species) of the studied plant species were found to reproduce only by seed, whilst 14 (23.0%) and 9 (14.8%) reproduce, in addition to by seed, by rhizome and seed and stolones propagule organs, respectively. In terms of species shoot-growth form, species with erect stem-growth form contributed over a third to the total species (42 species; 68.9%), whilst those with prostate and acaulescent growth forms, respectively, contributed 14 species (23.0%) and 5 species (8.2%) (for detail see Online Resource 1). Mean (± SE) FDis was 0.154 ± 0.003 (range: 0–0.25), whilst that of SES_FDis_ was −0.067 ± 0.068 (range: −1.83–2.76).

We counted a mean (± SE) number of 44.86 ± 3.67 (range: 0–316) and 30.76 ± 4.12 (range: 0–333) burrows plot^−1^ of root-rat old burrow and fresh burrow densities, respectively. Root-rat mima mounds were encountered at 97 plots, or 45% of the total plots. Livestock dung abundance varied between 0 and 250 (mean ± SE plot^−1^: 52.24 ± 3.70). Distance from human settlement ranged between 0 and 1250 m (mean ± SE: 625.00 ± 29.12).

**Fig. 2:**
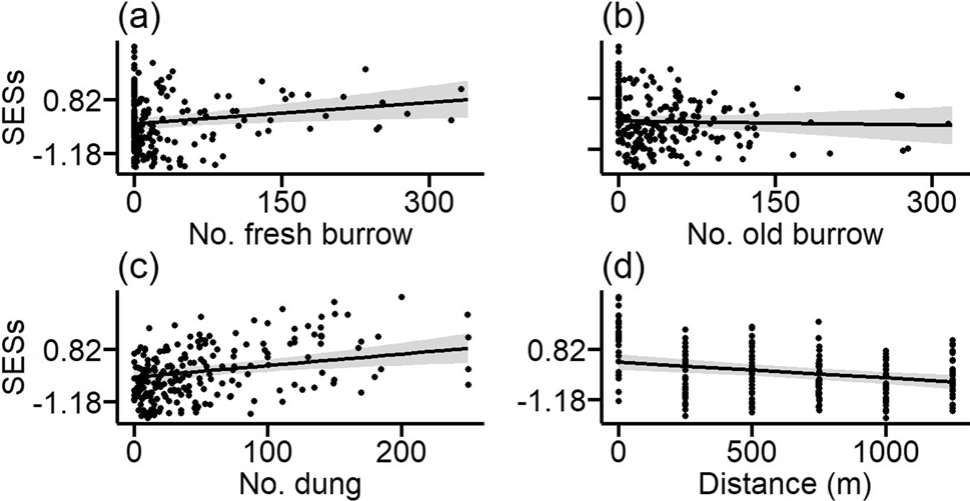
Relationships between disturbance by **a** the giant root-rat fresh burrow density (number plot^−1^) and **b** old burrow density (number plot^−1^) and human activities **c** livestock dung abundance (number plot^−1^) and **d** distance from settlement (m) with standardized effect size of (SESs) of overall traits

Testing if the observed SES_FDis_ of traits in each plot was higher or lower than expected from a null model revealed that mean SES_FDis_ was not significantly different from zero (*t* = −0.976, df = 215, *P* = 0.33). However, analysing the effects of giant root-rat and human disturbances on the FDis, we found significantly increased SES_FDis_ with increasing root-rat fresh burrow density (Z = 2.566, *P* < 0.05) and livestock dung abundance (Z = 3.121, *P* < 0.01), but lower at mima mound sites than at sites where mima mounds were absent (Z = 2.212, *P* < 0.05) and decreased with increasing distance from settlement areas (Z = 3.756, *P* < 0.001) (Table 1; Fig. 2a–d).

**Table 1:** Summary statistics for linear mixed-effects model testing the effects of human disturbances [distance from human settlement (m) and livestock dung abundance (no. plot^−1^)] and giant root-rat (*Tachyoryctes macrocephalus*) disturbances [giant root-rat fresh burrow density (no. plot^−1^), presence of mima mound, giant root-rat old burrow density (no. plot^−1^)] on standardized effect size (SES_FDis_) values

Explanatory variable	Estimate ± S.E	Z-value
Intercept	0.1484 ± 0.1957	0.754
Distance from settlement	−0.0006 ± 0.0002	3.756***
Livestock dung abundance	0.0045 ± 0.0014	3.121**
GRR fresh burrow density	0.0027 ± 0.0010	2.566*
Mima mound (present)	−0.2905 ± 0.1306	2.212*
GRR old burrow density	−0.0005 ± 0.0012	0.440

Asterisks indicate significance: *P < 0.05, **P < 0.01, ***P < 0.001

**Fig. 3:**
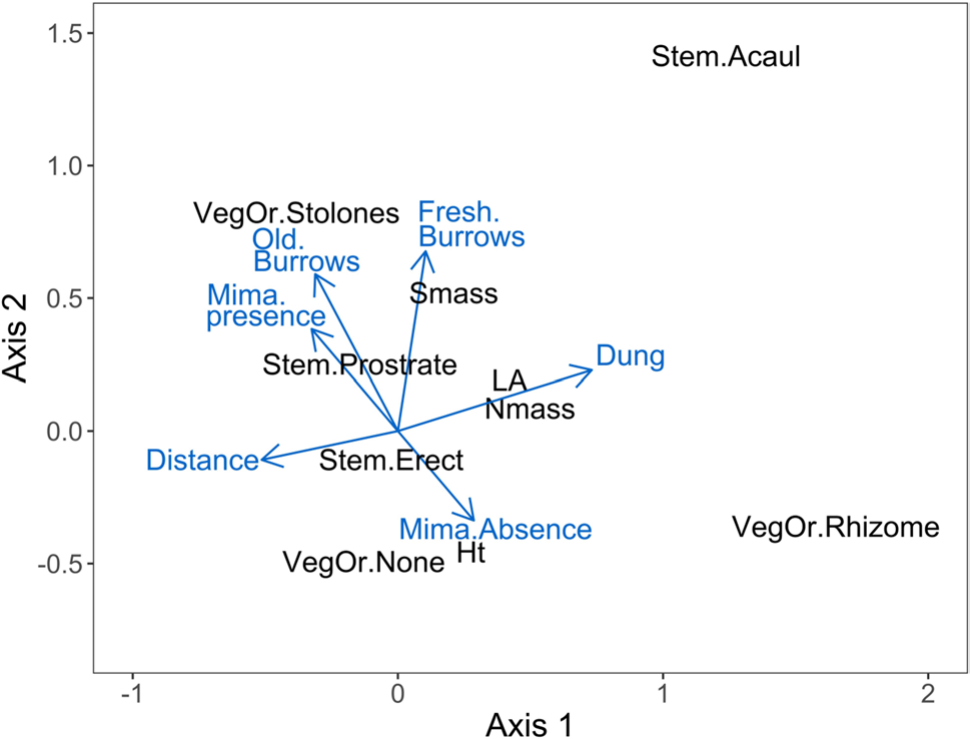
A biplot of associations between giant root-rat disturbance, human activities (livestock grazing and distance from settlement) and plant traits. Vectors depict the coefficients of the main dimensions along the first two axes of RLQ space. Abbreviations for disturbance variable: Mima.Presence = root-rat mima mound present; Mima.Absence = mima absent; Old.Burrows = root-rat old burrow density (no. plot^−1^); Fresh.Burrows = root-rat fresh burrow density (no. plot^−1^); Distance = distance from settlement (m); Dung = livestock dung density (no. plot^−1^). Abbreviations for traits: Veg.Or.None: no vegetative propagation; VegOr.Stolones = vegetation propagation via stolones; VegOr.Rhizome = vegetative propagation via rhizomes; Ht = plant adult height (m); Nmass = leaf nitrogen content (mg g^−1^); Smass = seed mass (mg); LA = leaf area (mm^2^); Stem.Acau = acaulescent stem shoot-growth form; Stem.Prosterate = prostrate stem-growth form; Stem.Erect = erect stem-growth form

Analyzing the multivariate link between species traits and environmental data, we found a significant general association between plant traits and disturbance measures (root-rat and human) than expected by chance (Monte-Carlo test, observed covariance: 0.578, expected covariance by chance: 0.231, *P* < 0.05). Results of the RLQ analysis showed that the variation in trait composition was explained by the disturbance data, with the first axis of the RLQ summarising most of the variation (eigenvalues: 0.55; covariance: 0.74; % total inertia: 87.7%), whilst the value for the second axis was: eigenvalues: 0.06; covariance: 0.24; %total inertia: 9.1% (Fig. 3). Correlation between the two new sets of factorial scores projected onto the first two RLQ axes was 0.41 and 0.18, respectively. Projected inertia, i.e. the amount of variance of each dataset captured by the Coinertia Analysis relative to the maximum possible value in the first two axes of the RLQ analysis, was 71.8% and 88.7% for the disturbances, and 62.4% and 71.3% for traits. Distance from human settlement and livestock dung abundance were associated with the first axis of the RLQ. Root-rat variables were associated with the second axis (Fig. 3).

**Fig. 4:**
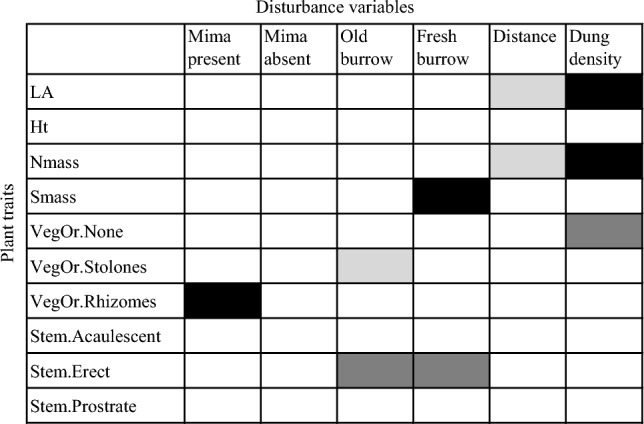
Fourth-corner analysis evaluating the associations of Afroalpine plant functional traits and giant root-rat engineering disturbance and human activity variables in the Bale Mountains, Ethiopia. Squares in black fill are significant positive ( +) associations at significance level of *P *< 0.05, grey fill significant negative associations at significance level of *P* < 0.05; and dark-grey fill marginal significant positive associations at *P* < 0.07. Abbreviations for disturbance variables, root-rat disturbances: Mima present = mima mound present; Mima absent = mima mound absent; Old burrow = root-rat old burrow density (no. plot^−1^); Fresh burrow = root-rat fresh burrow density (no. plot^−1^). Human disturbances: Distance = distance from settlement (m); Dung density = livestock dung density (no. plot^−1^). Abbreviations for plant traits: LA = leaf area (mm^2^); Ht = adult plant height (cm); Nmass = leaf nitrogen content (mg g^−1^); Smass = seed mass (mg); vegetative propagation organ type, VegOr.None = no vegetative propagation organ; VegOr.Stolones = propagation by seed and stolones; VegOr.Rhizomes = propagation by seed and rhizomes; stem shoot-growth form, Stem.Acaulescent = acaulescent stem shoot-growth form; Stem.Erect = Erect stem shoot-growth form; Stem.Prostrate = prostrate stem shoot-growth form

Examining disturbance specific associations with traits based on the fourth-corner analyses, we found significant associations of individual plant traits and disturbance variables (Figs. 3 and 4). Whilst increasing root-rat engineering disturbances were associated with larger seed mass, stoloniferous vegetative propagation and prostrate stem shoot, increasing human disturbances were associated, positively or negatively, with larger leaf size and increased leaf nitrogen content (Fig. 3). Specifically, increasing root-rat fresh burrows was positively associated with larger seed mass, root-rat old burrows negatively with rhizomatous vegetative propagation and presence of root-rat mima mound positively with stoloniferous vegetative propagation, whilst increasing livestock dung abundance was related to larger leaf size and increased leaf nitrogen content but increasing distance from settlement had the opposite relationship (Fig. 4). Certain species were found to be strongly associated with specific traits that in turn were associated with a specific disturbance type (Online Resource 5). For example, characteristic stolonifereous, prostrate rosette species, such as *Alchemilla abyssinica*, *Helichrysum gofense* and *Euryops prostrates*, were associated with root-rat mima mounds, and species such as *Crepis rueppelii, Haplocarpha rueppelii, Kniphofia foliosa, Potentilla dentate, Erigeron alpinus* and *Umbilicus bostryoides* with larger leaf size and higher nitrogen content were associated with human activities (Online Resource 5).

## Discussion

We identified associations between functional trait diversity and composition of Afroalpine plant communities and disturbances by giant root-rats and humans. Whilst we did not find an overall signal of FDis within our community, we found an increase in plant functional trait diversity with increasing root-rat (fresh burrows) engineering and increasing human activities. However, the diversity was lower at sites with root-rat mima mounds compared with sites where mima mounds were absent. Yet, each disturbance had specific associations with traits.

In line with our first prediction, both root-rat fresh burrows engineering disturbances and human activities (livestock grazing and settlement) appeared to enhance FDis, suggesting that these disturbances lead to increased habitat heterogeneity that, in turn, results in a functional trait divergence (Choler 2005). These disturbances are known to cause increased soil nutrient, create open spaces and reduce abundances of dominant species, which facilitate colonization and establishment of species with diverse traits (Choler 2005; Haussmann 2017). Particularly, the positive relationship of increasing soil nutrient––which is caused by livestock dung and urine, plant decompositions and soil mixing by root-rats––within increased plant functional diversity supports the notion that applications of organic fertilizers in agricultural landscapes promote the functional diversity of local weedy plant communities as it favours coexistence of species varying in their competitive response strategies to deal with different environmental conditions and management disturbances (Rotchés-Ribalta et al. 2023). In our study area, Šklíba et al. (2017) have reported that soils of giant root-rat mounds contain higher nitrogen and other nutrients, which could enhance coexistence of species with different trait values. This enhanced resource availability explains our finding of a significantly increased SES_FDis_ with increasing root-rat fresh burrow density (see Choler 2005; Escobedo et al. 2017; Boet et al. 2020). Similar studies (e.g. Chambers et al. 1990; Choler 2005; Asefa et al. 2022, 2023) have also reported the importance of disturbances from rodent engineering activities in maintaining or enhancing the structural and functional diversities of Arctic and Alpine meadow plant communities. Another effect of the disturbance by rodents that could explain the increase in functional diversity is that soil stir causes some seeds present in the seed bank to reach the surface and germinate, similar to the reports for wild boar (*Sus scrofa*) rooting disturbance promoting the germination of annuals present in the seed bank (Bueno et al. 2011; Ballari and Barrios-García 2014). Moderate level of disturbances [sensu Connell’s (1979) ‘Intermediate Disturbance Hypothesis’] caused by human activities, such as livestock grazing, often cause habitat heterogeneity and plant functional diversity, and thus have been used as management tool to improve rangeland conditions (Bokdam 2001; Eldridge et al. 2016; Pavlů et al. 2019). Grazing and trampling by livestock reduces plant biomass and creates open spaces for gap-colonizing plant species and increases soil nitrogen content from dung deposition and urination (Tessema et al. 2011; Eldridge et al. 2016; Niu et al. 2019; Pavlů et al. 2019). These livestock activities can enhance coexistence of species with different traits, resulting in enhanced FDis. In addition, through livestock dung and on their fur, new species might reach the community as seeds, which could lead to an increase in the functional diversity.

In the contrary to the above habitat heterogeneity process, but as predicted in our second hypotheses, our results showed three evidences that these disturbances also act as habitat filters. First, despite the positive relationship found with root-rat fresh burrow and human activities, the overall SES_FDis_ value was similar to that obtained from null model. Second, mean SES_FDis_ was lower at root-rat mima mounds, compared with at sites where mima mounds were absent. Finally, in line with our third prediction, we found disturbance specific associations with traits (see below). In the first case, such mean value of SES_FDis_ near zero can be the outcome of two assembly processes, which may off-set each other’s effects: random species distribution (no effect of disturbances) vs. concurrent occurrence of competition, or habitat heterogeneity, and habitat filtering processes (Edwards et al. 2021; de Bello et al. 2021). Different disturbance types influence specific assembly process and/or trait differentially (Choler 2005; Wang et al. 2020; Smith et al. 2022); for example, removal of plant biomass via feeding and burrowing activities of root-rats create space and increase nutrient level at root-rat fresh burrow, thereby causing habitat heterogeneity; whilst its mima mound acts as habitat filter, in line with our second prediction. The latter finding can be explained by root-rat’s long-term activities at mima mounds that may allow persistence of species only adapted to such frequent long-term disturbances. Thus, the lack of significant departure of mean SES_FDis_ from zero in our community can likely be attributed to the simultaneous operation of habitat heterogeneity and habitat filtering assembly processes, which leads their resultant effect at plot level to be stable functional trait diversity or mean SES_FDis_ value approaching zero (de Bello et al. 2021; Smith et al. 2022).

In line with our third prediction, we found disturbance specific associations with traits. Considering root-rat disturbance, for example, we found strong association of larger seed mass with increasing root-rat fresh burrow density (positive association), rhizomatous vegetative propagation with increasing giant root-rat old burrow (negative), and stolonifereous vegetative propagation with presence of giant root-rat mima mound (positive) (Fig. 4). These results indicate that different engineering disturbance types of subterranean rodents to have varying filtering effects on biodiversity and ecosystem functioning (see also Jones et al. 1997; Tallents 2007; Asefa et al. 2022, 2023) and that considering different types of disturbances would help reveal the mechanisms how engineering activities of rodent impact plant functional trait diversity. More specifically, the filtering effect of the giant root-rat mima mound, for example, can be explained in relation to previous studies who reported strong associations with mima mounds of characteristic stolonifereous, prostrate rosette species, such as *Alchemilla abyssinica*, *Helichrysum gofense* and *Euryops prostratus* (Beyene 1986; Miehe and Miehe 1994; Tallents 2007; see also Fig. 3 and Online Resource 5). The latter two species are endemic to the Bale Mountains and have been recorded only at giant root-rat mima mound sites (Miehe and Miehe 1994; Tallents 2007; Asefa et al. 2022, 2023). Overall, the stolonifereous trait is amongst commonly known traits of alpine plants that serve as a reproduction strategy to reduce resource allocation to seed production (Choler 2005). Thus, as mima mounds are relatively elevated compared with the surrounding areas (Wraase et al. 2023), being stoloniferous enables such characteristic species to persist on mima mounds by overcoming alpine wind pressure. Our finding of positive association of seed mass with root-rat fresh burrow density can be explained by the fact that this disturbance favours species with large seed size as large seeds facilitate survival through the early stages of recruitment, and higher establishment in the face of rodent herbivory and burrowing disturbances (Westoby et al. 2002; Muller-Landau 2010). Similar to our finding, Šklíba et al. (2017) have reported strong association of plant species with relatively larger seed mass, such as *Urtica simensis,Carduus nyassanus* and *Salvia merjamie*, with giant root-rat fresh mounds (Figs. 3 and 4). The former two species are also characterized by mechanical defence against herbivores (Šklíba et al. 2017), in line with our third prediction and similar to the high abundance of unpalatable plants observed in the Tibetan plateau as a consequence of the activity of the plateau zokors (*Myospalax baileyi*; Zhang and Liu 2003; Wang et al. 2020). The negative association between old burrow density and rhizomatous propagation and why fresh burrows and old burrows are associated to different functional strategies can be explained by the mechanisms how root-rat engineering activities (burrowing and foraging) lead to mosaic of vegetation successions (Jones et al. 1997). Root-rats destroy either the whole plant biomass or only aboveground or belowground parts of the biomass. It is thus poss ible that plants with rhizomatous vegetative organs might be damaged more due to underground digging and foraging although their aboveground parts may still be alive at fresh burrows (Beyene 1986; Yaba et al. 2011). However, such plants may die out by the time the fresh burrows are abandoned (transformed to old age) or are suppressed by other plants, resulting to them to be filtered out.

Similarly, larger leaf size and leaf nitrogen content are strongly associated with human activities (Figs. 3 and 4). This result is in line with our third prediction and corroborates findings of previous studies that human activities related to livestock production and human settlements are globally known to favour plants with resource-acquisitive traits (Grime 2001; Wright et al. 2004; Dunne et al. 2011). Higher leaf area and leaf nitrogen content are widely known as indicative of a fast-growing rapid nutrient acquisition strategy (Smith et al. 2022), as the combination of these traits increases energy exploitation through improved photosynthetic capacity (Wright et al. 2004). Overall, the increased levels of leaf area and leaf nitrogen content with increasing livestock grazing intensity and with decreasing distance from human settlements permits the success of quick-growing plants that can quickly access resources (Bokdam 2001; Eldridge et al. 2016; Niu et al. 2019; Pavlů et al. 2019; Smith et al. 2022). Despite the missing of some important leaf traits and grass species in our dataset, particularly leaf mass area and leaf dry matter content, which may potentially affect the interpretation of our results, our findings suggest the association of plants with acquisitive leaf economics spectrum with human activities. Characteristic species with such acquisitive leaf economics spectrum associated with human activities include: *Crepis rueppelii, Haplocarpha rueppelii, Kniphofia foliosa, Potentilla dentate* and *Umbilicus bostryoides*. Most of these species have relatively larger leaf area (see Online Resource 1 and 3) and are known to be weedy growing in degraded areas (see Online Resource 2).

In conclusion, we found overall stable functional trait diversity, which likely is due to opposing effects (creation of habitat heterogeneity vs habitat filtering) of the different disturbance types and associations of each disturbance type with specific trait types. Our results also highlight that even in the case of a disturbance caused by the same animal (rodent) species, the type of disturbance (mima mounds, old burrows, fresh burrows) selects for or against specific and different traits or strategies. This is revealed by our findings of strong positive association of larger seed mass with increasing root-rat fresh burrow density, rhizomatous vegetative propagation negatively with increasing giant root-rat old burrow, and stolonifereous vegetative propagation positively with presence of giant root-rat mima mound. However, human disturbances associated with increasing grazing intensity filtered species with larger leaf area and higher leaf nitrogen content whilst increasing distance from settlement filtered species with smaller leaf area and lower leaf nitrogen content. From these findings, it is possible to conclude that two-plant strategies can be revealed: traits related to survival and reproduction strategies are associated with root-rat disturbances, while leaf traits related to resource-acquisitive economics spectrum are associated with human disturbances (Díaz et al. 2016). In sum, identification of such associations between plant traits and disturbance can help predicting changes under future environmental change and clarifying on which trait-disturbance associations to focus in effective ecosystem management.

### Supplementary Information

Below is the link to the electronic supplementary material.

Supplementary file1 (PDF 287 KB)

## Data Availability

All relevant data are within the manuscript and its Supplementary Material.

## Code availability

R code used for analysis is provided in Electronic Supplementary Material.
