# Supplementary material for: Giant root-rat engineering and livestock grazing activities regulate plant functional trait diversity of an Afroalpine vegetation community in the Bale Mountains, Ethiopia
Source: Oecologia. 2024 Jun 1;205(2):281–93. doi: 10.1007/s00442-024-05563-6 (PMC11281956; doi:10.1007/s00442-024-05563-6)
Supplement: Supplementary file 1 — Supplementary file1 (PDF 287 KB) [file 442_2024_5563_MOESM1_ESM.pdf]

Electronic Supplemental Material (ESM)

Giant root-rat engineering and livestock grazing activities alter plant functional trait composition of an Afroalpine vegetation community in the Bale Mountains, Ethiopia

Addisu Asefa[a], Victoria Reuber[a], Georg Miehe[b], Luise Wraase[c], Tilaye Wube[d], Nina Farwig[a] and Dana G. Schabo[a]

[a]Conservation Ecology, Department of Biology, Philipps-Universität Marburg, Karl-vonFrisch-Straße 8, 35043 Marburg, Germany

[b]Vegetation Geography, Department of Geography, Philipps-Universität Marburg, Deutschhausstraße 10, 35032 Marburg, Germany

[c]Environmental Informatics, Department of Geography, Philipps-Universität Marburg, Deutschhausstraße 12, 35032 Marburg, Germany

[d]Department of Zoology, College of Natural and Computational Sciences, Addis Ababa University, Po Box 1176, Addis Ababa, Ethiopia.

Corresponding author: Addisu Asefa, Conservation Ecology, Department of Biology, Philipps-Universität Marburg, Karl-von-Frisch-Straße 8, 35043 Marburg, Germany; email: aa.mitiku@gmail.com

Online Resource 1. Species-specific trait values used in this study.

Species name	Species code	Leaf area, (LA; mm^2)	Leaf Nitrogen (Nmass; mg/g)	Height (Ht; cm)	Seed mass (Smass; mg)	Vegetative organ (VegOrg)	Stem shoot growth form
Agrocaria melanantha	AG.MEL	1932.98	22.829	70	0.8924	Rhizome	Erect
Alchemilla abyssinica	AL.ABY	2784.38	14.48	6	0.36	Stolones	Erect
Alchemilla microbetula	AL.MIC	358.25	10.28	6	0.61	Stolones	Prostrate
Alchemilla pedata	AL.PED	1437.75	12.34	15	0.36	Stolones	Prostrate
Anagallis serpens	AN.SER	98.75	25.1795	40	0.49	Stolones	Prostrate
Anthemis tigreensis	AN.TIG	236.075	25.76	60	0.522	None	Erect
Artemisia abyssinica	AR.ABY	446.84	30.6067	60	0.035	None	Erect
Artemisia afra	AR.AFR	110.55	17.8667	100	0.21	None	Erect
Arabis alpina	AR.ALP	297.85	28.6554	60	0.27575	None	Erect
Arabis thaliana	AR.THA	59.16	54.1618	25	0.046	None	Erect
Cardamine hirsuta	CA.HIR	405	22.8574	45	0.10711	None	Erect

Carduus nyassanus	CA.NYA	18144	47.92	150	14.77	None	Erect
Cardamine obliqua	CA.OBL	2493.75	41.92	120	0.54	Rhizome	Erect
Cerastium afromontanum	CE.AFR	71.389	22.7164	75	0.21	None	Prostrate
Cineraria abyssinica	CI.ABY	8.04	17.9	100	0.51	None	Erect
Conium maculatum	CO.MAC	24310	54.82	250	2.73257	Rhizome	Erect
Crepis rueppelli	CR.RUP	3840	18.3531	35	0.11	None	Acaulescent
Cotula abyssinica	CT.ABY	143.775	25.76	20	0.21513	None	Erect
Cynoglossum lanceolatum	CY.LAN	1147.5	12.8638	70	4.2908	None	Erect
Dispsacus pinnatifidus	DI.PIN	4593.75	13.1	300	4.41	Rhizome	Erect
Erigeron alpinus	ER.ALP	243.311	25.5386	50	0.216	Rhizome	Acaulescent
Erophila verna	ER.VER	339.739	25.3737	9.5	0.0235	None	Erect
Euryops prostratus	EU.PRO	7.2	15.1	9	2.06731	Stolones	Prostrate
Galium acrophyllum	GA.ACR	5.6	27.088	30	3.01	Stolones	Prostrate
Geranium arabicum	GR.ARA	23.29	24.8039	10	2.1184	Stolones	Prostrate
Gnaphalium unions	GN.RUB	52.5	24.5	40	0.0345	Stolones	Prostrate
Hedbergia abyssinica	HE.ABY	2961	22.2539	300	0.06764	None	Erect

Haplocapha rueppelii	HA.RUP	2199.38	20.96	13	0.87	Rhizome	Acaulescent
Helichrysum citrispinum	HE.CIT	7.455	20.91	75	0.05	None	Erect
Helichrysum cymosum	HE.CYM	916.988	28.8	120	0.05	None	Erect
Hebenstretia angolensis	HE.DET	5651	24.25	105	0.92	None	Erect
Helichrysum forsskahlii	HE.FOR	31.725	19.43	75	0.1	None	Erect
Helichrysum gofense	HE.GOF	47.025	20.91	15	0.06	Stolones	Prostrate
Helichrysum splendidum	HE.SPL	53.475	8.05	75	0.03	None	Erect
Kniphofia foliosa	KN.FOL	15439.5	24.25	40	1.6855	Rhizome	Erect
Lythrum rotundifolium	LY.ROT	65.625	25.72	30	0.30833	None	Erect
Malva verticillata	MA.VER	40095	43.424	300	1.97	None	Erect
Myocotes keniensis	MY.KEN	11.3953	24.795	5	0.3	Rhizome	Erect
Plantago afra	PL.AFR	79.313	7.53385	50	0.96575	None	Erect
Polygonum afromontanum	PO.AFR	85.27	27.955	100	1.29	None	Prostrate
Potentilla dentata	PO.DEN	933.8	27.5431	40	0.4	Rhizome	Acaulescent
Ranunculus multifidus	RA.MUL	2317.78	8.4	40	1.55	None	Erect
Ranunculus oreophytus	RA.ORE	119.025	28.4781	40	2.44	None	Erect

Ranunculus stagnalis	RA.STA	284.274	26.4	33	1.82	None	Erect
Rumex nepalensis	RU.NEP	5025	22.32	100	2.21	None	Erect
Salvia merjamie	SA.MER	2854.22	10.56	100	1.79716	None	Erect
Satureja pseudosimensis	SA.PSE	32.663	17.2	100	0.67	None	Erect
Satureja punctata	SA.PUN	26.584	35.728	35	0.04	None	Erect
Scabiosa columbaria	SC.COL	1206	20.5355	45	1.884	Rhizome	Erect
Sedum baleensis	SE.BAL	27.335	24	5	0.03	None	Acaulescent
Sedum mooneyi	SE.MOO	10.988	24.0289	10	0.04453	None	Prostrate
Senecio schultzii	SE.SCH	2486.59	25.45	30	0.445	None	Prostrate
Senecio unionis	SE.UNI	66.816	25.45	35	0.455	None	Erect
Sonchus melanolepis	SO.MEL	681.6	30.06	45	9.30081	None	Prostrate
Swertia abyssinica	SW.ABY	367.413	24.3657	65	0.06	None	Erect
Swertia kilimangerica	SW.KIL	944.7	21.9869	150	0.1928	None	Erect
Trifolium acaule	TR.ACA	13.02	38.2986	5	1.34	None	Prostrate
Umbilicus botryoides	UM.BOT	5670	25.47	38	5.79	Rhizome	Erect
Urtica simensis	UR.SIM	2571.25	28.1412	100	52	Rhizome	Erect

Veronica abyssinica	VE.ABY	462.3	17.5	40	0.05	Rhizome	Erect
Veronica glandulosa	VE.GLA	151.725	16.06	50	0.07	None	Erect

Online Resource 2. List of references from which plant species height, leaf area size and morphological data were extracted.

References

a) Flora of Ethiopia and Eritrea

Edwards S, Demissew S, Hedberg I (1997) Flora of Ethiopia & Eritrea, Volume 6: Hydrocharitaceae to Arecaceae. The National Herbarium, Addis Ababa, Ethiopia and Uppsala, Sweden.

Edwards S, Tadesse M, Hedberg I (eds.) (1995) Flora of Ethiopia and Eritrea Volume 2, Part 2: Canellaceae to Euphorbiaceae. The National Herbarium, Addis Ababa, Ethiopia, and Uppsala, Sweden.

Edwards S, Tadesse M, Demissew S, Hedberg I (eds.) (2000) Flora of Ethiopia & Eritrea, Volume 2, Part 1: Magnoliaceae to Flacourtiaceae. The National Herbarium, Addis Ababa, Ethiopia and Uppsala, Sweden.

Hedberg I, Edwards I (eds.) (1989) Flora of Ethiopia, Volume 3: Pittosporaceae to Araliaceae. The National herbarium, Addis Ababa and Asmara, Ethiopia, and Uppsala, Sweden.

Hedberg I, Edwards S, Nemomissa S (eds.) (2003) Flora of Ethiopia and Eritrea Volume 4, Part 1: Apiaceae to Dipsacaceae. The National herbarium, Addis Ababa, Ethiopia, and Uppsala, Sweden.

Hedberg I, Friis I, Edwards S (eds.) (2004) Flora of Ethiopia and Eritrea, Volume 4, Part 2: Asteraceae (Compositae). The National Herbarium, Addis Ababa, Ethiopia, and Uppsala, Sweden.

Hedberg I, Kelbessa E, Edwards S, Demissew S, Persson E (eds.) (2006) Flora of Ethiopia and Eritrea Volume 5: Gentianaceae to Cyclocheilaceae. The National Herbarium, Addis Ababa University, Addis Ababa, Ethiopia, and Uppsala, Sweden.

b) Other references from which traits were extracted

Adamu, E.; Asfaw, Z.; Demissew, S.; Baye, K. 2022. Proximate, Minerals, and Vitamin C Contents of Selected Wild Edible Plants in Lasta District, Northeastern Ethiopia. Int. J. Plant Biol. 2022, 13, 613–624. https://doi.org/10.3390/ijpb13040049.

Costa, D.S., Zotz, G., Hemp, A. and Kleyer, M. 2018. Trait patterns of epiphytes compared to other plant life-forms along a tropical elevation gradient. Functional Ecology. 2018;32:2073–2084. DOI: 10.1111/1365-2435.13121.

Dagem Alemayehu, Gulelat Desse, Kebede Abegaz, Beruk Berhanu Desalegn, Dereje Getahun. 2016. Proximate, Mineral Composition and Sensory Acceptability of Home Made Noodles from Stinging Nettle (*Urtica simensis*) Leaves and Wheat Flour Blends. International Journal of Food Science and Nutrition Engineering, 6(3): 55-61. DOI: 10.5923/j.food.20160603.02

Ishfaq Hameed and Ghulam Dastagir. 2009. Nutritional analyses of *Rumex hastatus* D. Don, *Rumex dentatus* Linn and *Rumex nepalensis* Spreng. African Journal of Biotechnology 8(17):4131-4133.

Koju Bedekach Bayba, Amare Aregahegn Dubale, Bewketu Mehari and Minale Shewa Atlabachew. 2020. Chemical Composition of *Urtica simensis* Grown in Different Regions of Ethiopia. Hindawi Journal of Chemistry Volume 2020, Article ID 9546178, 8 pages.

Mekonnen, B., Zech, W., Glaser, B., Lemma, B., Bromm, T., Nemomissa, S., Bekele, T., and Zech, M.: Chemotaxonomic patterns of vegetation and soils along altitudinal transects of the

Bale Mountains, Ethiopia, and implications for paleovegetation reconstructions – Part 1: stable isotopes and sugar biomarkers, E& G Quaternary Sci. J., 68, 177–188, https://doi.org/10.5194/egqsj-68-177-2019, 2019

Metin Turan, Saban Kordali, Hüseyin Zengin, Atilla Dursun & Yıldırım Sezen 2003. Macro and Micro Mineral Content of Some Wild Edible Leaves Consumed in Eastern Anatolia, Acta Agriculturae Scandinavica, Section B - Plant Soil Science, 53:3, 129-137, DOI: 10.1080/090647103100095.

Moges, E. & Balakrishnan, M. 2014. Nutritional composition of food plants of geladas (*Theropithecus gelada*) in Guassa Community Protected Area, Ethiopia. Journal of Biology, Agriculture and Healthcare, 4, 23, 38-44.

Plumptre, A.J. 1995. The chemical composition of montane plants and its influence on the diet of the large mammalian herbivores in the Parc National des Volcans, Rwanda. J. Zool. Lond. 235:323-337.

Zewudu, G. and Dalle, G. 2019. Evaluation of Nutritive Value of Some Native Forage Species in Tikur Incinni District, Oromia, Ethiopia. Forage Res., 45 (2):103-110.

Online Resource 3. List of plant species, their height, leaf dimensions, leaf shape and leaf area estimation. For details and abbreviations, see methods section in the main text. MP = Montemgory parameter for leaf shape types: c = a correction factor to account for differences in leaf shape type.

Species name	Species code	Height max (cm)	Leaf length (L, cm)	Leaf width, (W, cm)	Leaf shape	L*W	MP, c	Leaf area, (cm2; = c*L*W*)
Agrocaria melanantha	AG.MEL	70	8.25	3.30	linear-lanceolate	27.23	0.71	19.33
Alchemilla abyssinica	AL.ABY	6	8.25	4.50	reniform-orbicular,	37.13	0.75	27.84
Alchemilla microbetula	AL.MIC	6	2.5	2.30	ovate	5.75	0.67	3.85
Alchemilla pedata	AL.PED	15	4.5	4.50	oblong-elliptic	20.25	0.71	14.38
Anagallis serpens	AN.SER	40	1.25	1.00	obovate	1.25	0.79	0.99
Anthemis tigreensis	AN.TIG	60	3.5	0.95	oblong or elliptic	3.33	0.71	2.36
Artemisia abyssinica	AR.ABY	60	2.5825	2.58	ovate	6.67	0.67	4.47
Artemisia afra	AR.AFR	100	5.5	0.30	ovate	1.65	0.67	1.11

Arabis alpina	AR.ALP	60	3.7	1.15	spathulate to oblong	4.26	0.70	2.98
Arabis thaliana	AR.THA	25	1.45	0.60	spathulate to elliptic,	0.87	0.68	0.59
Cardamine hirsuta	CA.HIR	45	6.75	0.80	ovate to orbicular	5.40	0.75	4.05
Cardamine obliqua	CA.OBL	120	9.5	3.50	elliptic to suborbicular	33.25	0.75	24.94
Carduus nyassanus	CA.NYA	150	27	10.50	oblanceolate	283.50	0.64	181.44
Cerastium afromontanum	CE.AFR	75	2.325	0.45	Ovate to lanceolate	1.03	0.69	0.71
Cineraria abyssinica	CI.ABY	100	3	0.04	ovate	0.12	0.67	0.08
Conium maculatum	CO.MAC	250	20	17.00	oblong-lanceolate	340.00	0.72	243.10
Cotula abyssinica	CT.ABY	20	2.25	0.90	linear	2.03	0.71	1.44
Crepis rueppelli	CR.RUP	35	20	3.00	oblanceolate	60.00	0.64	38.40
Cynoglossum lanceolatum	CY.LAN	70	7.5	2.13	lanceolate, oblong-Ianceolate or linearlanceolate,	15.94	0.72	11.48
Dispsacus pinnatifidus	DI.PIN	300	12.5	5.25	lanceolate	65.63	0.70	45.94

Erophila verna	ER.VER	9.5	1.3	3.79	obovate, spatulate, oblanceolate, lanceolate, oblong	4.92	0.69	3.40
Erigeron alpinus	ER.ALP	50	5.25	0.65	linear-oblong or elongate-lanceolate	3.41	0.71	2.43
Euryops prostratus	EU.PRO	15	0.8	0.13	oblong-linear,	0.10	0.72	0.07
Galium acrophyllum	GA.ACR	30	0.7	0.13	(ob)lanceolate	0.09	0.64	0.06
Geranium arabicum	GR.ARA	10	0.85	0.40	ovate - lanceolate	0.34	0.69	0.23
Gnaphalium unions	GN.RUB	40	2.5	0.30	spathulate, obtuse or subacute	0.75	0.70	0.53
Haplocapha rueppelii	HA.RUP	13	7.5	4.25	elliptical	31.88	0.69	21.99
Hebenstretia angolensis	HE.DET	50	3.375	0.24	linear or linear-lanceolate;	0.80	0.71	0.57
Hedbergia abyssinica	HE.ABY		2.1	0.20	elliptic to lanceolate	0.42	0.71	0.30
Helichrysum citrispinum	HE.CIT	75	0.7	0.15	linear-Ianceolate or oblong	0.11	0.71	0.07
Helichrysum cymosum	HE.CYM	120	9.5	1.35	oblong-lanceolate	12.83	0.72	9.17
Helichrysum forsskahlii	HE.FOR	75	1.2	0.38	lanceolate or linear	0.45	0.71	0.32

Helichrysum gofense	HE.GOF	15	2.375	0.28	oblong-linear, linear or linear-Ianceolate	0.65	0.72	0.47
Helichrysum splendidum	HE.SPL	75	1.5	0.50	linear-oblong or Ianceolate	0.75	0.71	0.53
Kniphofia foliosa	KN.FOL	40	60	3.65	linear lanceolate	219.00	0.71	154.40
Lythrum rotundifolium	LY.ROT	30	1.25	0.75	obovate to orbicular or oblong-elliptical,	0.94	0.70	0.66
Malva verticillata	MA.VER	300	27	27.00	cordiform or reniform	729.00	0.55	400.95
Myocotes keniensis	MY.KEN	5	1.875	0.09	oblong or lanceolate	0.16	0.72	0.11
Plantago afra	PL.AFR	50	4.5	0.25	linear or linear-lanceolate;	1.13	0.71	0.79
Potentilla dentata	PO.DEN	40	5.6	2.30	oblong to lanceolate	12.88	0.73	9.34
Ranunculus multifidus	RA.MUL	100	6.75	5.13	ovate	34.59	0.67	23.18
Ranunculus oreophytus	RA.ORE	40	1.5	1.15	Elliptic	1.73	0.69	1.19
Ranunculus stagnalis	RA.STA	33	1.65	3.13	lobed	5.17	0.55	2.84
Rumex nepalensis	RU.NEP	100	12.5	6.00	ovate	75.00	0.67	50.25
Salvia merjamie	SA.MER	100	10.5	3.90	oblong, elliptic or ovate	40.95	0.70	28.54
Satureja pseudosimensis	SA.PSE	35	0.75	0.65	ovate	0.49	0.67	0.33

Satureja punctata	SA.PUN	100	0.85	0.45	circular to ovate	0.38	0.70	0.27
Scabiosa columbaria	SC.COL	70	9	2.00	ovate or obovate	18.00	0.67	12.06
Sedum baleensis	SE.BAL	0.5	1.1	0.35	linear	0.39	0.71	0.27
Sedum mooneyi	SE.MOO	1	0.41	0.40	obovate	0.16	0.67	0.11
Senecio schultzii	SE.SCH	30	15.5	2.25	Iinear-oblong to oblanccolate	34.88	0.71	24.87
Senecio unionis	SE.UNI	35	5.8	0.16	linear or oblong	0.93	0.72	0.67
Sonchus melanolepis	SO.MEL	45	6	1.60	linear	9.60	0.71	6.82
Swertia abyssinica	SW.ABY	65	3.25	1.70	oblanceolate, elliptic	5.53	0.67	3.67
Swertia kilimangerica	SW.KIL	150	6	2.35	obovate	14.10	0.67	9.45
Trifolium acaule	TR.ACA	5	0.7	0.30	obcordate to oblanceolate	0.21	0.62	0.13
Umbilicus botryoides	UM.BOT	38	9	9.00	peltate, ± circular, concave	81.00	0.70	56.70
Urtica simensis	UR.SIM	100	8.5	5.50	subcordate	46.75	0.55	25.71
Veronica abyssinica	VE.ABY	40	3	2.30	ovate	6.90	0.67	4.62
Veronica glandulosa	VE.GLA	50	2.125	1.05	ovate to elliptic	2.23	0.68	1.52
Polygonum afromontanum	PO.AFR	100	2.5	0.50	ovate-elliptic	1.25	0.68	0.85

Online Resource 4: All R scripts used for analysis in this study

```
#Load packages
library(ade4)
library(picante)
library(DHARMa)
library(performance)
library(ggplot2)
library(glmmTMB)
library(cowplot)
```

#Load data

#Data description: Three datasets were use – 1) Disturbance (5 predictors); 2) SppTrait (6 species traits); and 3) SppAbundance (cover abundances of 61 alpine plant species recorded from 216 plots across 5 sites in the Bale Mountains of Ethiopia)

#Predictor variables: Mima = root-rat mima mound; Old_m = giant root-rat old burrow density; Fresh_m = giant root-rat fresh burrow density; Dung = number of cow dung; Distance (m) from human settlement

#SppTraits: adult maximum height (Ht; cm); leaf size (LA, mm2); leaf nitrogen content (Nmass; mg.g), seed mass (mg); Vegetative organ (seed alone, seed and rhizome, seed and stolones); stem growth form (Acaulescence [Acaul.], Erect, Prostrate)

#A: conduct functional trait diversity analysis

```
#A1: compute Functional trait diversity (functional trait dispersion; FDis)

#A1.1: first calculate gowdis dissimilarity for species trait variables

gowdisTrait <- gowdis(SppTrait, SppAbundance) #Spp refers to species


#A1.2: calculate FDis

FDis <- fdisp(gowdisTrait, SppAbundnace, tol = 1e-07)

#A2: estimate the standardized effect size (SES) of FDis

SESFDis <- ses.mntd(SppAbundance, TraitDissimilarity, null.model = "independentswap",

abundance.weighted=FALSE, runs = 999, iterations = 1000)

summary(SESFDis)

print(SESFDis)


#A3: Conduct t-test to see whether mean SES of FDis was significantly different from zero

t.test(FDCWMR$SES, mu = 0, alternative = "two.sided")


#A4: analyse the relationship between SES of FDis and predictor variables using GLMM

SESFDisglmm <- glmmTMB(SES ~ Mima + Old_m +Fresh_m + Dung +Dist

+(1|site/transect), na.action = na.fail, family = gaussian(), data = FDCWMR)


#A4.2a: test model assumptions using residual plots

testDispersion(SESFDisglmm) #dispersion is fine

testZeroInflation(SESFDisglmm) #No problem detected

simulationFDis <- simulateResiduals(fittedModel = SESFDisglmm, plot = F)
```

```
plot(simulationFDis)#plot(simulationS_norm)
plotResiduals(simulationFDis, form = FDCWMR$Fresh_m)
plotResiduals(simulationFDis, form = FDCWMR$Dist)
plotResiduals(simulationFDis, form = FDCWMR$Dung) #Quantile deviation detected
plotResiduals(simulationFDis, form = FDCWMR$Old_m)
plotResiduals(simulationFDis, form = FDCWMR$Mima)

#A4.2b: test model assumptions using multicollinearity test
check_collineairty(SESFDisglmm) # no collinearity problem was detected

#A4.2c: check fit of the full model by comparing with reduced models using AIC
mavSES <- dredge(SESFDisglmm, options(na.action = "na.fail"))
print(mavSES) #Full model was ranked 2nd with 0.018 deltaAIC from the top best model
#Thus, we used the full model for parameter estimation

#A5: Obtain parameter estimates
summary(SESFDisglmm)
print(SESFDisglmm)

#A6: create plots of response against quantitative predictors
FDisfreshm_plot<-plot(ggpredict(FDisRF5glmmEsES, terms=c("Fresh_m")) %>%
plot(rawdata=T, dot.alpha = 1, dot.size=10, color = "black")+#, limit.range=T) +
labs(x="No. fresh burrow", y="SES", title="a")+
```

```
theme_bw() + theme(panel.grid.major = element_blank(),
panel.grid.minor = element_blank(),
panel.border = element_blank(),
axis.line = element_line(colour = "black", size=4),
axis.text = element_text(colour = "black", size=48),
title=element_text(size=48), legend.text = element_text(size=90))
+
scale_y_continuous(breaks=seq(-1.18,2.77,1.95))+
scale_x_continuous(breaks = seq(0,330,150)) +
geom_line(size=4))#,face="bold"
FDisoldm_plot<-plot(ggpredict(FDisRF5glmmEsES, terms=c("Old_m")) %>%
plot(rawdata=T, dot.alpha = 1, dot.size=10, color = "black")+#, limit.range=T) +
labs(x="No. old burrow", y=NULL, title="b")+
theme_bw() + theme(panel.grid.major = element_blank(),
panel.grid.minor = element_blank(),
panel.border = element_blank(),
axis.line = element_line(colour = "black", size=4),
axis.text = element_text(colour = "black", size=48),
axis.text.y = element_blank(),
title=element_text(size=48), legend.text = element_text(size=90)) +
scale_y_continuous(breaks=seq(-1.18,2.77,1.95))+
scale_x_continuous(breaks = seq(0,330,150)) +
annotation_logticks(mid = unit(3, "mm")) +
```

```
geom_line(size=4))#,face="bold

FDisdung_plot<-plot(ggpredict(FDisRF5glmmEsES, terms=c("Dung")) %>%

plot(rawdata=T, dot.alpha = 1, dot.size=10, color = "black")+#, limit.range=T) +

labs(x="No. dung", y="SES", title= "c")+

theme_bw() + theme(panel.grid.major = element_blank(),

panel.grid.minor = element_blank(),

panel.border = element_blank(),

axis.line = element_line(colour = "black", size=4),

axis.text = element_text(colour = "black", size=48),

title=element_text(size=48), legend.text = element_text(size=90)) +

scale_y_continuous(breaks=seq(-1.18,2.77,1.95))+

scale_x_continuous(breaks = seq(0,250,100)) +

annotation_logticks(mid = unit(-3, "mm")) +

geom_line(size=4))#,face="bold

FDisdist_plot<-plot(ggpredict(FDisRF5glmmEsES, terms=c("Dist")) %>% plot(rawdata=T,

dot.alpha = 1, dot.size=10, color = "black")+#, limit.range=T) +

labs(x="Distance (m)", y=NULL, title= "d") +

theme_bw() + theme(panel.grid.major = element_blank(),

panel.grid.minor = element_blank(),

panel.border = element_blank(),

axis.line = element_line(colour = "black", size=4),

axis.text = element_text(colour = "black", size=48),

axis.text.y = element_blank(),
```

```
title=element_text(size=48), legend.text = element_text(size=90)) +
scale_y_continuous(breaks=seq(-1.18,2.77,1.95))+
scale_x_continuous(breaks = seq(0,1250,500)) +
geom_line(size=4))#,face="bold

#A7: create a grid of all plots
pgridall <- plot_grid(FDisfreshm_plot, FDisoldm_plot, FDisdung_plot, FDisdist_plot,
align = "hv", nrow=2, ncol = 2) +
theme(plot.margin = unit(c(-0.2, 0, -0.6, 0), "cm"))
#B: conduct RLQ and fourth-corner analyses
#B1: RLQ - first conduct separate ordinations of three tables (TableL, TableR, TableQ)
OrdSppAbundL <- dudi.coa(SppAbundance, scannf = FALSE, nf = 2)
OrdEnvironmentR <- dudi.hillsmith(SiteEnvironment,scannf = FALSE, nf = 2, row.w =
OrdSppAbundL6$lw)
OrdSppTraitQ <- dudi.hillsmith(SppTrait, scannf = FALSE, nf = 2, row.w =
OrdSppAbundL$cw)

#B2: conduct RLQ analysis
RLQplant <- rlq(OrdEnvironmentR, OrdSppAbundL, OrdSppTraitQ, scannf = FALSE, nf =
2)
summary(RLQplant)
summary(OrdSppAbundL)
```

summary(OrdEnvironmentR)

summary(OrdSppTraitQ)

#B3: Print 'rlq'

print(RLQplant)

##B4: plot ‘rlq’

plot(RLQplant, xax = 1, yax = 2)

##B5: obtain and print summary ‘rlq’

RLQsmry <- summary(RLQplant)

print(RLQsmry)

##B6: Evaluate RLQ significance of the global, or their joint multivariate relationship, between the trait-environment relationships

RLQplantRT <- randtest(RLQplant,nrepet = 999, modeltype = 6)

summary(RLQplantRT)

print(RLQplantRT)

#B7: create biplots of trait vs environment, and trait vs species

biplot(RLQplant$c1, RLQplant$l1, xlab = "Axis 1", ylab = "Axis 2")

biplot(RLQplant$lQ, RLQplant$c1, xlab = "Axis 1", ylab = "Axis 2")

#B8: Conduct 4th corner analysis

veg4thC <- fourthcorner(SiteEnvironment, SppAbundance, SppTrait, modeltype = 6, nrepet = 999, tr01 = FALSE)

#B9: Obtain p adjusted values

veg4thCpadj <- p.adjust.4thcorner(veg4thC, p.adjust.method.G = p.adjust.methods, p.adjust.method.D = p.adjust.methods, p.adjust.D = "levels")

summary(veg4thC)

print(veg4thC)

summary(veg4thCpadj)

print(veg4thCpadj)

#B10: use fourth.corner2 function to evaluate the global, or their joint multivariate relationship, significance of the traits-environment

veg4thC2 <- fourthcorner2(SiteEnvironment, SppAbundance, SppTrait, modeltype = 6, nrepet = 999, tr01 = FALSE)

veg4thC2padj <- p.adjust.4thcorner(veg4thC2, p.adjust.method.G = p.adjust.methods, p.adjust.method.D = p.adjust.methods, p.adjust.D = "levels")

summary(veg4thC2)

summary(veg4thC2padj)

plot(veg4thC, alpha = 0.05, stat = "D2")

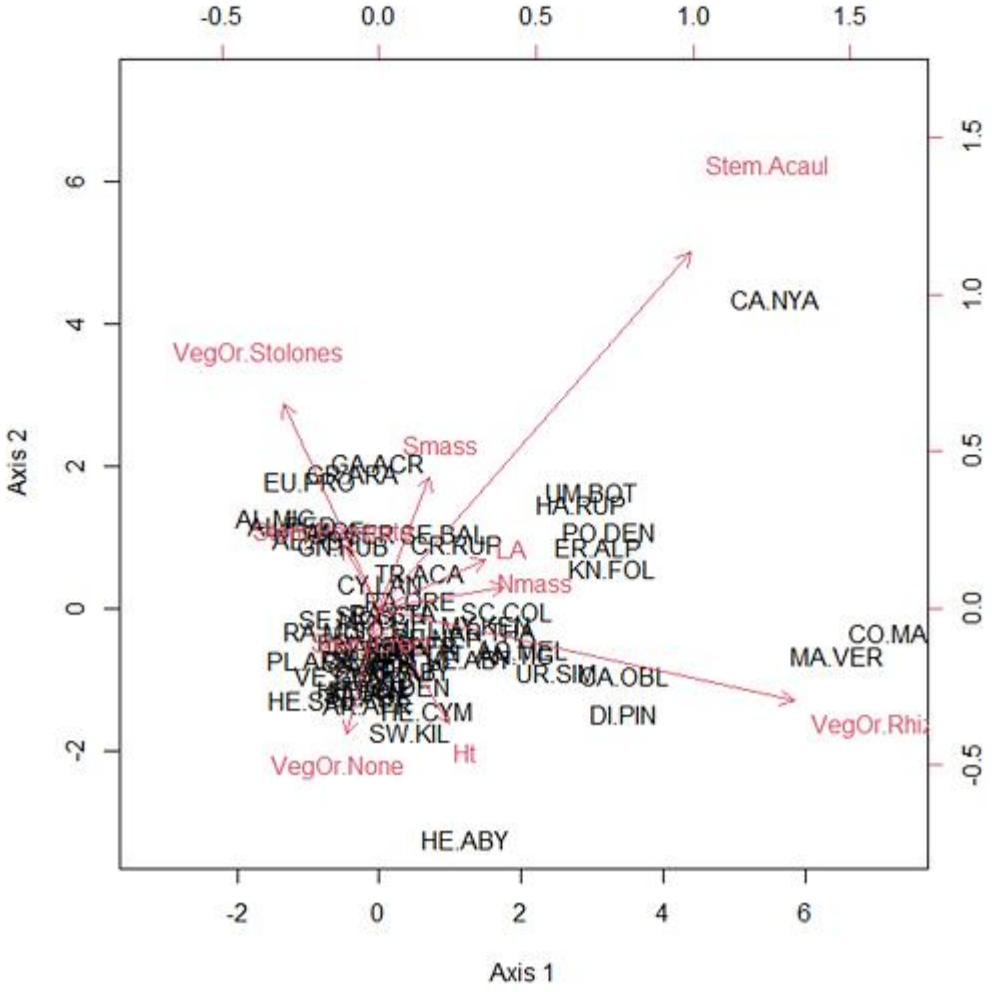

Online Resource 5. A biplot of associations between plant species and traits. Vectors depict the coefficients of the main dimensions along the first two axes of RLQ space. Full names of plant species are provided in the Online Resource 3. Abbreviations for traits: VegOr.None: no vegetative propagation; VegOr.Stolones = vegetation propagation via stolones; VegOr.Rhizome = vegetative propagation via rhizomes; Ht = plant adult height; Nmass = leaf nitrogen content; Smass = seed mass; LA = leaf area; Stem.Acau = acaulescent stem shoot growth form; Stem.Prostrate = prostrate stem growth form; Stem.Erect = erect stem growth form.
